# Optimizing Microfluidic Impedance Cytometry by Bypass Electrode Layout Design

**DOI:** 10.3390/bios14040204

**Published:** 2024-04-19

**Authors:** Guangzu Wu, Zhiwei Zhang, Manman Du, Dan Wu, Junting Zhou, Tianteng Hao, Xinwu Xie

**Affiliations:** 1 Systems Engineering Institute, Academy of Military Sciences, People’s Liberation Army, Tianjin 300161, China; gzzzzw@163.com (G.W.); zhangzhiwei0124@163.com (Z.Z.); 2 National Bio-Protection Engineering Center, Tianjin 300161, China; 3 School of Environmental Science and Engineering, Tianjin University, Tianjin 300072, China; dumanman@tju.edu.cn; 4 School of Electronic Information and Automation, Tianjin University of Science and Technology, Tianjin 300222, China; wuxiaozhenzhuzi@163.com (D.W.); dianxinzjt@163.com (J.Z.); haotianteng2001@163.com (T.H.)

**Keywords:** microfluidic impedance cytometry, no bypass electrode, floating electrode, grounding electrode, sensing sensitivity

## Abstract

Microfluidic impedance cytometry (MIC) has emerged as a popular technique for single-cell analysis. Traditional MIC electrode designs consist of a pair of (or three) working electrodes, and their detection performance needs further improvements for microorganisms. In this study, we designed an 8-electrode MIC device in which the center pair was defined as the working electrode, and the connection status of bypass electrodes could be changed. This allowed us to compare the performance of layouts with no bypasses and those with floating or grounding electrodes by simulation and experiment. The results of detecting Φ 5 μm beads revealed that both the grounding and the floating electrode outperformed the no bypass electrode, and the grounding electrode demonstrated the best signal-to-noise ratio (SNR), coefficient of variation (CV), and detection sensitivity. Furthermore, the effects of different bypass grounding areas (numbers of grounding electrodes) were investigated. Finally, particles passing at high horizontal positions can be detected, and Φ 1 μm beads can be measured in a wide channel (150 μm) using a fully grounding electrode, with the sensitivity of bead volume detection reaching 0.00097%. This provides a general MIC electrode optimization technology for detecting smaller particles, even macromolecular proteins, viruses, and exosomes in the future.

## 1. Introduction

Traditional methods for detecting cells typically capture the characteristics of the entire cell population, potentially overlooking important details about specific subsets and rare samples. Consequently, there has been growing interest among researchers in single-cell detection techniques. Single-cell analysis is highly significant in biochemical measurements and medical research, such as cell classification [1], cancer diagnosis [2], as well as subpopulation analysis of bacteria and cells that require more comprehensive and refined single-cell data.

Fluorescence-activated cell sorting (FACS) is a widely utilized technique for single-cell detection that enables automated analysis and sorting of cells [3]. However, FACS requires professional and skilled technicians and staining and electrostatic deflection can affect the viability and original physiological status of cells [4]. Additionally, the expensive and bulky optical equipment of the instrument limits its development in point-of-care testing (POCT) and areas with limited medical resources [5]. Consequently, many researchers have focused on microfluidic impedance cytometry (MIC) technology owing to its advantages, such as being label-free, low-cost, easy to operate, and having minimal sample consumption. With advancements in microelectronics and mechanical system (MEMS) technology, MIC has been widely used in cell counting [6], volume identification [7], and cell viability classification [8].

It is crucial to achieve sensitive and accurate cell detection results in MIC [9]. Various optimization techniques have been developed to enhance the performance of MIC platforms for bio-particle event detection, including improving the electrode layout [10,11,12,13], channel design [14,15,16,17], and data processing algorithms [18,19]. These optimizations aim to reduce the baseline noise, decrease the coefficient of variation (CV) of events, increase the signal-to-noise ratio (SNR) of particle events, and improve the detection capability of the MIC system. Electrode layout designs include coplanar, facing, extruded (3D structure), and liquor electrodes [20]. Electrode configurations have been optimized by several researchers by incorporating floating or grounding electrodes among the coplanar and facing electrodes. Daniel et al. utilized grounding electrode pairs in the middle and bypass of the two facing working electrode pairs, respectively. The time spans of both the pulse peaks of the working electrode and grounding electrode were used to calculate the vertical position of a bio-particle in the channel and calibrate its size [21,22]. Additionally, the amplitude peaks of an event can be used to calibrate the particle size. For example, the particle size was calculated based on changes in current compared to a standard particle [23], and the ratio of the amplitude peaks of an event at different positions fitted the electrical size of the particles [22,24,25,26]. Ninno et al. applied floating electrodes between the gaps of three coplanar working electrodes, which generated bipolar double-Gaussian bio-particle events and reduced the CV of bead events [24]. Ai et al. added floating electrodes between double differential electrodes, resulting in the generation of a trough at the floating electrode. Finally, the bio-particle size was calculated using a numerical fitting method, and submicron particles (400 nm) were measured [27]. Fang et al. utilized a coplanar three-electrode design with two floating and tilting electrodes and completed the calibration of particles in the tube space [28]. Additionally, Federica et al. integrated floating and grounding electrodes in the middle and on both sides for the facing differential electrode pairs to generate a bipolar Mexican-hat pulse particle event, which can be used as a bio-particle event for feature recognition, thereby mitigating false-positive occurrences [29]. Zhou et al. used a similar electrode layout, in which all electrodes except for the working electrode pairs were designed as grounding electrodes, effectively reducing the current crosstalk between adjacent facing electrodes. The simulation demonstrated that the change in the impedance value increased approximately five-fold for different particle sizes [30]. Xie et al. designed a MIC system with six floating electrodes and a working electrode pair to achieve high SNR events and a low coincidence rate [31].

Numerous researchers have focused on enhancing the precise and consistent detection of particle events within the same species using these methods. In most studies, floating or grounding electrodes are used to increase the peak and trough of a particle event [21,23,24,27,28,32]. Currently, these two electrodes are mainly utilized for calibrating the flow position of particles in the channel to eliminate pulse difference caused by the variations in electric field strength at the height of the coplanar electrode or current inhibition from the ipsilateral electrode of the facing differential electrode pair [30,32,33]. In addition, pulses from the floating and grounding electrodes were employed as features for particle identification. However, no systematic studies have been conducted on the influence of floating and ground electrodes on MIC systems. Furthermore, most previous studies connected these two types of electrodes between working electrodes, which leads to an increased probability of detecting coincident particles because of the expanded detection area.

In this study, we designed and tested a simple, practical, and universal coplanar electrode design that does not require additional components. The main focus was to compare and evaluate the performance of a pair of coplanar working electrodes with and without bypass electrodes, including floating and grounding electrode layouts and varying grounding electrode areas. The comparison parameters included the SNR of the event, baseline noise, and CV of the amplitude and phase. The best detection was achieved by using a fully grounded electrode layout. Therefore, the optimized electrode layout was employed to detect small beads in a wide channel, and the particle detection sensitivity of the system was calculated. The proposed method can also be utilized to detect particles of various sizes, offering a universal optimization technique for coplanar electrodes for future investigations of biological protein macromolecules, viruses, and bacteria.

## 2. Materials and Methods

### 2.1. Sample Preparation

Polystyrene beads (Φ 0.7, 1, 2, 3, and 5 μm, Duke Standards 3000/4000 series) were purchased from Thermo Fisher Scientific (Waltham, MA, USA), with all original size CV of 1%, and concentrations of 1 × 10^9^, 1.0 × 10^9^, 0.88 × 10^9^, 0.33 × 10^9^, 0.042 × 10^9^ particles/mL, respectively. Polydimethylsiloxane (PDMS) and cross-linking agent (SYLGARD 184) were purchased from Dow Corning (Midland, MI, USA). Bovine serum albumin (BSA) and phosphate-buffered saline (PBS) were purchased from Solarbio Life Sciences (Beijing, China). The beads were suspended and diluted in a 3% BSA solution (a mixture of 0.3 mg BSA and 10 mL PBS). The BSA solution and bead suspension medium were stored at 4 °C.

### 2.2. Electrode Design, Chip Fabrication, and the MIC System Setup

#### 2.2.1. Electrode Design

**Figure 1:**
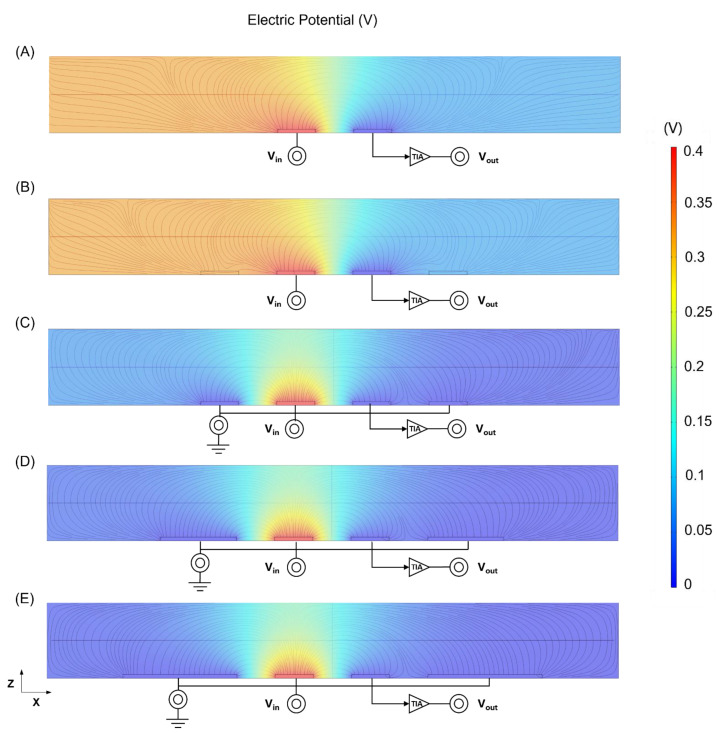
The electric field simulation of different electrode layouts. (**A**) No bypass electrode state. (**B**) Bypass floating electrode state. (**C**) Bypass grounding electrode state (width: 10 μm). (**D**) Bypass grounding electrode state (width: 20 μm). (**E**) Bypass grounding electrode state (width: 30 μm).

The electric field simulation of the three bypass states and different bypass grounding electrode areas was shown in Figure 1A–E by using COMSOL Multiphysics (COMSOL Inc., Stockholm, Sweden).

**Figure 2:**
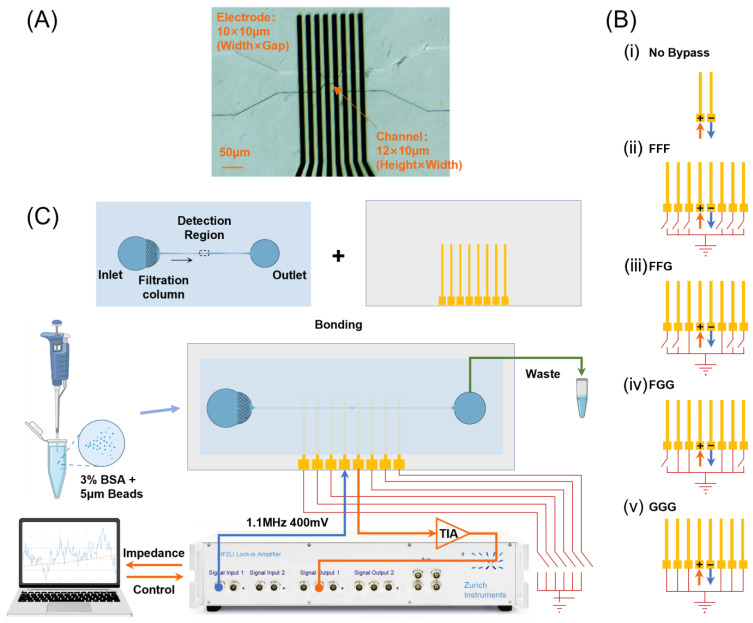
The design of an 8-electrode MIC chip and the whole system. (**A**) Optical microscopy of the detection region captured using a CCD camera. (**B**) Five electrode layouts; (**i**) no Bypass; (**ii**) FFF; (**iii**) FFG; (**iv**) FGG; (**v**) GGG. (**C**) Schematic diagram of microfluidic electrical impedance cytometry.

As shown in Figure 2A, the sensing region was captured by a CCD camera (Motion BLITZ EoSens Cube7, Mikrotron, Munich, Germany). The conventional coplanar electrode layout in the MIC system consists of a single pair of working electrodes, as shown in Figure S1. The sensing region for particle detection is primarily concentrated between the two middle electrodes; however, electric field leakage may still occur [23]. The current in the circuit loop flows towards a component with a lower impedance, such as bypass electrodes. Therefore, current leakage was suppressed, and the sensing performance was improved. As shown in Figure 2A, we designed a chip with eight independent electrodes and set four different electrode layouts to compare their performance with the traditional two-electrode layout design (seen in Figure S1). As shown in Figure 2C, it shows only the sensing electrode status (no bypass) and different combinations of floating and grounding electrodes (FFF, FFG, FGG, and GGG, respectively). Different electrode layout designs can be easily switched from one to another using wire jumpers (similar to switches) on the printed circuit board (PCB) of the MIC device. This design allows the comparison of different electrode layouts using one device to avoid the differences between the electrodes or PDMS channels caused by the fabrication processes. Additionally, three separate electrode pairs connected to the same end (grounding or floating), rather than a wider electrode, can reduce the risk of liquor leakage. The electric field simulation of this 8-electrode design is shown in Figure S2.

#### 2.2.2. Chip Fabrication

The MIC chip consisted of a PDMS channel (volume proportion of PDMS and cross-linking agent: 10:1), a glass chip with gold electrodes, and a PCB. This channel was fabricated using common soft lithography technology, the details of which can be found in previous studies [8,15]. The electrode substrate was fabricated using photolithography and lift-off techniques and comprised two layers: a 20 nm thick titanium (Ti) layer on the glass and a 200 nm thick gold (Au) layer on top of the titanium. The MIC chip was fabricated by bonding the channel and electrode substrate using a plasma cleaner (CPC-B; CIF International Group Co., Ltd., Beijing, China) under air conditions. The bonding process involved applying a power of 100 W and an RF time of 15 s. After aligning the working electrode pair and the detection area of the PDMS channel (as depicted in Figure 2A,B), the chip was dried at 50 °C for 30 min in a vacuum drying oven (DZF6050; Supo Instrument Co. Ltd., Zhejiang, China). The impedance chip channel size was 12 × 150 μm (height × width) and contracted to 12 × 10 μm at the detection area, and the electrode substrate size was 10 × 10 μm (width × gap).

#### 2.2.3. MIC System Setup

After injecting the bead suspension medium into the microfluidic channel inlet, it passed through the filtration columns within the channel. An impedance pulse is generated when a particle passes through a coplanar working electrode pair in the detection region. The impedance data were amplified using a current amplifier (HF2TA; Zurich Instruments, Zurich, Switzerland) and a locked-in amplifier (HF2FI; Zurich Instruments, Zurich, Switzerland). Finally, the impedance signals were uploaded to a computer for display and storage purposes. The excitation signal frequency was set to 1.1 MHz with an amplitude of 400 mV, and the sampling rate f_s_ of the impedance signal was set to 1.799 kHz.

### 2.3. Experiment Design and Procedures

#### 2.3.1. Comparison of Different Bypass Electrodes

To investigate the influence of floating and grounding bypass electrodes, the Φ 5 μm beads were passed through the sensing region of the MIC device using different electrode layouts (no bypass, FFF, and GGG, respectively). The channel size was 12 × 10 (height × width), and the electrode size was 10 × 10 μm (width × gap), as depicted in Figure 2A. The Φ 5 μm beads were suspended in the 3% BSA medium, diluted 50 times, and a 40 μL sample was added into the inlet. The liquid level difference between the inlet and outlet was approximately 6 mm for controlling the flow rate. After injecting the bead suspension medium into the inlet, the resistance, reactance, and impedance were measured and displayed on a master computer (Labone; Zurich Instruments, Zurich, Switzerland). The signal was not recorded until a stable state was reached. After conducting the tests for approximately 15 min and closing the recording, the sample inlet was cleaned with BSA solution. When no bead event appeared within one one-minute window, both the bead suspension medium and electrode configuration were changed simultaneously for the next measurement.

#### 2.3.2. Comparison of Different Areas of Grounding Bypass Electrode

To investigate the influence of the grounding electrode area on impedance sensing, different electrode layouts (FFF, FFG, FGG, and GGG) of the same device were tested using the same chip and beads under the same conditions. The initial concentration was calculated by the supplier, using a specified method. The concentration of the bead medium was determined and calculated using microscopic and hemocytometer analysis.

#### 2.3.3. Detection Capability of the Optimized Electrode Layout

To test the capability of the optimized electrode layout, a channel with the size of 12 × 150 μm (height × width) was employed, and beads with varying sizes (Φ 0.7, 1, 2, 3, and 5 μm) were detected, respectively. The bead concentration, inlet liquid volume, and data recording and analysis methods were unchanged.

### 2.4. Data Acquisition and Analysis Methods

The original resistance ($Z_{\mathrm{RE}}$) and reactance ($Z_{\mathrm{IM}}$) were amplified and acquired using a lock-in amplifier and data acquisition (DAQ) board, which were stored in the computer. Data processing was designed and implemented in MATLAB (Math Works, Natick, MA, USA), including loading and integrating the dataset, recognizing bio-particle events, computing and storing characteristic parameters and analyzing data features. Particle event recognition utilizes the central limit theorem [34], baseline tracking, and peak value recognition methods to calculate the event peak values of $Z_{\mathrm{RE}}$ and $Z_{\mathrm{IM}}$ after accounting for drift with the baseline. Characteristic parameter amplitude (Z) and phase (θ) of impedance signal are then calculated as

(1) $$\left|Z\right|=\sqrt{\Delta Z_{\mathrm{RE}}^{2}+\Delta Z_{\mathrm{IM}}^{2}},$$

(2) $$\Delta \theta =\arctan\frac{\Delta Z_{\mathrm{RE}}}{\Delta Z_{\mathrm{IM}}},$$

(3) $${\Delta Z}_{X}=V_{\mathrm{peak}}-V_{\mathrm{baseline}},$$

where $V_{\mathrm{peak}}$ represents the actual pulse amplitude of the event and $V_{\mathrm{baseline}}$ is the actual amplitude of the baseline noise. ${\Delta Z}_{X}$ is the pulse amplitude of a bead event, and X represents the real (RE) and imaginary (IM) part of the impedance.

The event noise range of the baseline (Noise), average SNR of all bead events, and CV are given by

(4) $$\mathrm{Noise}=\sqrt{{\left(3\times {\sigma_{Z}}_{\mathrm{RE}}\right)}^{2}+{\left(3\times {\sigma_{Z}}_{\mathrm{IM}}\right)}^{2}}$$

(5) $$\mathrm{SNR}=20{\;\times \;\log}_{10}\frac{\Delta \left|Z\right|}{\mathrm{Noise}},$$

(6) $$\mathrm{CV}=\frac{\sigma_{x}}{{\mathrm{Mean}}_{x}},$$

where ${\sigma_{Z}}_{\mathrm{RE}}$, ${\sigma_{Z}}_{\mathrm{IM}}$, and $\sigma_{x}$ represent the standard deviation of $Z_{\mathrm{RE}}$, $Z_{\mathrm{IM}}$, and x (Z or θ), ${\mathrm{Mean}}_{x}$ is the mean value of x (amplitude and phase).

Coplanar sensing electrodes cannot fully capture particles because of the disparity between the particle size and channel height. The coefficient of channel loss k (≥0) is obtained by comparing it with the actual particle concentration [15], and is as follows:

(7) $$k=1-\frac{C_{\mathrm{meas}}}{C_{\mathrm{hemocytometer}}},$$

where $C_{\mathrm{meas}}\;\mathrm{and}\;C_{\mathrm{hemocytomer}}$ are the mean concentration measured by MIC and hemocytometer (Huida Medical Instrument Co., Ltd., Yancheng, China), respectively.

Therefore, we compared the calculated initial concentrations and hemocytometer results with the measured particle concentrations using different electrode layouts. The bead concentration, measured using the MIC, was calculated by dividing the number of particles passing through the detection area in one minute by the volume of the detection area:

(8) $$C_{\mathrm{meas}}=\frac{N}{V}\times D,$$

(9) $$V=W_{d}\times H_{d}\times \nu {\times T}_{m},$$

(10) $$\nu =\frac{L_{d}}{W_{e}/T_{s}},$$

where N is the number of particles flowing through the detection region in one minute, and $C_{\mathrm{meas}}$ is the measured concentration of the particle suspension medium. V is the volume of liquid flowing through the detection area in one minute, $W_{d}$ denotes the width of the detection region (10 μm), $H_{d}$ refers to its height (12 μm). The variable ν is the liquid flow rate (μm/s), and $T_{m}$ = 60 s. Additionally, $L_{d}$ indicates the length of the detection region (~40 μm), $W_{e}$ is the width of bead events (sample point counts; $W_{e}$ = 13.72, 13.03, 12.72, and 18.95 for FFF, FFG, FGG, and GGG electrode layout, respectively), and $T_{s}=1/f_{s}$.

The particle volume detection sensitivity was defined as [35]:

(11) $$\mathrm{Sensitivity}=\frac{V_{p}}{V_{\mathrm{detection}\;\mathrm{region}}},$$

where $V_{p}$ and $V_{\mathrm{detection}\;\mathrm{region}}$ are the volume of particles and detection region. The length of detection represents the length of the detection region as the width of two detection electrodes plus the gap between the detection electrodes.

All experiments were repeated three times, and the data were processed with statistical significance analysis using the *t*-test.

## 3. Results and Discussion

### 3.1. The Comparison among No Bypass, Floating and Grounding Electrode Layout

#### 3.1.1. Electric Field Simulation Result of Different Bypass Electrode Statuses

As shown in Figure 1A–C, a comparison between no bypass and bypass electrodes revealed that the addition of bypass electrodes effectively converges the electric field and mitigates its leakage. The bypass floating electrode also had the effect of less charge leakage than the no bypass electrode in Figure S3. Furthermore, these electrodes can reduce circuit impedance and shorten the electrical loop length, resulting in a lower background noise range. Additionally, as illustrated in Figure 1B,C, compared with the floating electrode state, the ground electrode can shield the electrical signal changes outside the detection area, and the disturbance caused outside the ground electrode directly flows into the ground electrode. And the electric potential outside the working area was almost zero, so the noise was further reduced.

#### 3.1.2. Statistical Results of Beads Detection

**Figure 3:**
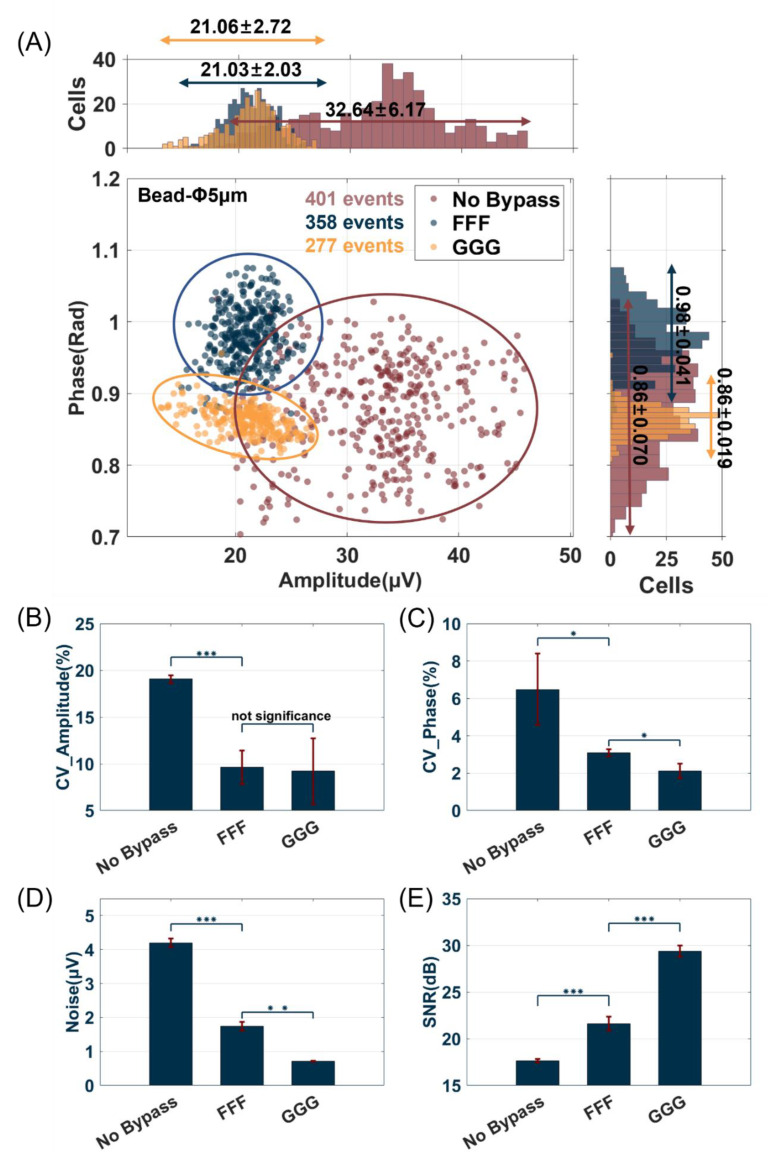
Comparison of statistical results of three electrode layouts (no bypass, FFF, and GGG). (**A**) Scatter plot and histogram of all bead events of amplitude and phase. Four event quality evaluation parameters with CV of amplitude (**B**) and phase (**C**), Noise (**D**), and SNR (**E**). (Error bars indicate standard deviation, *n* = 3, * *p* < 0.1, ** *p* < 0.01, *** *p* < 0.001).

To investigate the influence of floating and grounding bypass electrodes, the channel size of this experiment was fixed to 12 × 10 (height × width), as shown in Figure 2A. The statistical results of detecting beads (Φ 5 μm) with different electrode layouts (each with ~350 events) are shown in Figure 3A. Compared with the no bypass group, both the FFF and GGG groups exhibited a decrease in the impedance amplitude, as depicted in the scatter plot and histogram in Figure 3A. The electrical signal generated in the detection area will primarily be directed towards the negative electrode of the working electrode while leading to a reduction in the overall electrical signal due to partial current flows towards the bypass electrode. However, the scatter plot results of FFF and GGG demonstrated a higher cluster degree than that of no bypass, with GGG showing the highest level of clustering.

The data distributions of the bead events for the three different electrode layouts are shown in Figure 3B–E. As illustrated in Figure 3B,C, the CV of amplitude decreased remarkably (*p* < 0.001) from no bypass (19.0667 ± 0.3963%) to FFF (9.6267 ± 1.8151%) and GGG (9.2100 ± 3.5343%), with no statistically significant difference observed between the FFF and GGG (*p* = 0.8647). In terms of phase, there were significant differences (*p* < 0.1) in the CV among no bypass (6.4867 ± 1.9041%), FFF (3.0933 ± 0.1872%), and GGG (2.1167 ± 0.3853%). Therefore, when evaluating bead cluster degree based on both amplitude and phase measurements, it can be calculated that the GGG electrode layout demonstrated the most pronounced improvement compared to other layouts. Although there was no significant difference in amplitude between FFF and GGG, the improvement in the CV in the phase (*p* < 0.1) of GGG can help better distinguish different particles, such as dead and live bacteria [8,9].

As shown in Figure 3D,E, the baseline noise and average SNR of the no bypass, FFF, and GGG groups demonstrated significant differences. The FFF and GGG electrode layouts reduced the noise amplitude, but the SNR increased. So, we used the range value of the baseline noise as a characteristic parameter. When no particles pass through the microfluidic channel, the impedance signal primarily comprises Gaussian white noise, which serves as the baseline noise. The range of this noise is calculated as three times the standard deviation (±3 σ) of the impedance signal (Noise), and the SNR and CV are determined using Equations (4)–(6). The baseline noise of the electrode status of no bypass (4.1967 ± 0.1250 μV) can be suppressed by floating (FFF, 1.7433 ± 0.1305 μV, *p* < 0.001) or grounding (GGG, 0.7147 ± 0.01 μV, *p* < 0.001) electrodes bypass, approximately reduced by a factor of 2.4 and 5.9, respectively. There was an overall increase in the average SNR, from no bypass (17.6367 ± 0.1861 dB) to FFF (21.6167 ± 0.7575 dB) or GGG (29.3667 ± 0.5914 dB), representing an increase of about 4 and 7 dB, respectively (for all groups, *p* < 0.001). A comparison between the GGG and no bypass electrode layouts reveals an approximately two-fold decrease in the CV of the amplitude and a three-fold decrease in the CV of the phase. Comparing the GGG and no bypass electrode layouts showed an improvement of over 10 dB in the average SNR for all bead events and a reduction in the noise effect by approximately 5.9 times. These experimental results were consistent with the simulation results, and the bypass grounding electrode had the best detection performance. The corresponding processing data are listed in Tables S1 and S2.

#### 3.1.3. Shape Characteristics of the Event Pulse

**Figure 4:**
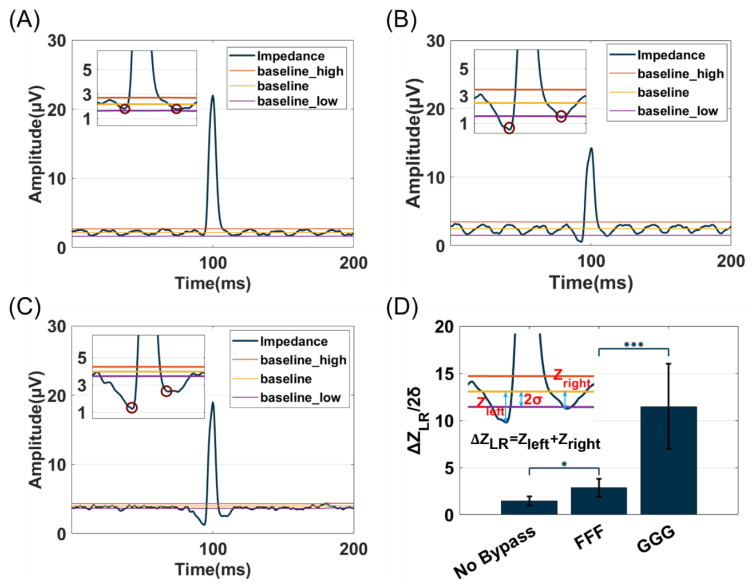
Comparison of event reverse characteristics of no bypass (**A**), FFF (**B**), and GGG (**C**) electrode layout (the ‘impedance’ curve represents the original impedance signal, the ‘baseline’ indicates the average background signal, the ‘baseline_high’ and ‘balseline_low’ represent ±2σ of background signal, and red circles represent reverse amplitude peak positions). (**D**) The average intensity of bead events of reverse feature position (ΔZ_LR_ is the sum of peak reverse plus values from left ($Z_{\mathrm{left}}$) and right (Z_right_) positions subtracted baseline; Error bars indicate the standard deviation, * *p* < 0.1, *** *p* < 0.001, *n* = 10).

As shown in Figure 4A–C, event pulses with distinct shapes are generated when using different electrode layouts. The reverse pulse signals are generated on both sides of the forward pulse of particles in FFF and GGG groups, forming a ‘W’ shape known as Mexican-hat [29] and can serve as an event characteristic for particle identification to reduce false positives in particle event recognition. $\Delta Z_{\mathrm{LR}}$ is calculated by summing left ($Z_{\mathrm{left}}$) and right (Z_right_) peak reverse pulse value (subtracting mean background noise baseline), while σ represents the standard deviation of background noise. Since the noise range is mostly set as twice the background noise standard deviation, $\Delta Z_{\mathrm{LR}}/2\sigma$ is used as the intensity parameter of the reverse pulse feature. As shown in Figure 4D, an analysis of 10 events of each group demonstrated that the $\Delta Z_{\mathrm{LR}}/2\sigma$ of both no bypass (1.4692 ± 0.4905 dB) and FFF (2.8522 ± 0.9590 dB) is less than 6 dB (6 dB is the minimum value when ΔZ_LR_ > 2 × 2σ) and thus cannot be recognized as a ‘Mexican-hat’ shape. While that of GGG (11.4863 ± 4.5151 dB) demonstrates a significant increase (*p* < 0.001) compared to FFF and exhibits a typical ‘Mexican-hat’ shape, indicating that the GGG electrode layout produces larger reverse pulses, making it more suitable for particle event identification.

### 3.2. Influence of Bypass Grounding Electrode Area

The wider the area of the grounding electrode simulation, as illustrated in Figure 1C–E, the greater the shielding effect can be achieved. Furthermore, the electric field simulation of the different bypass floating electrode areas was shown in Figure S3, and there was no obvious difference between them.

**Figure 5:**
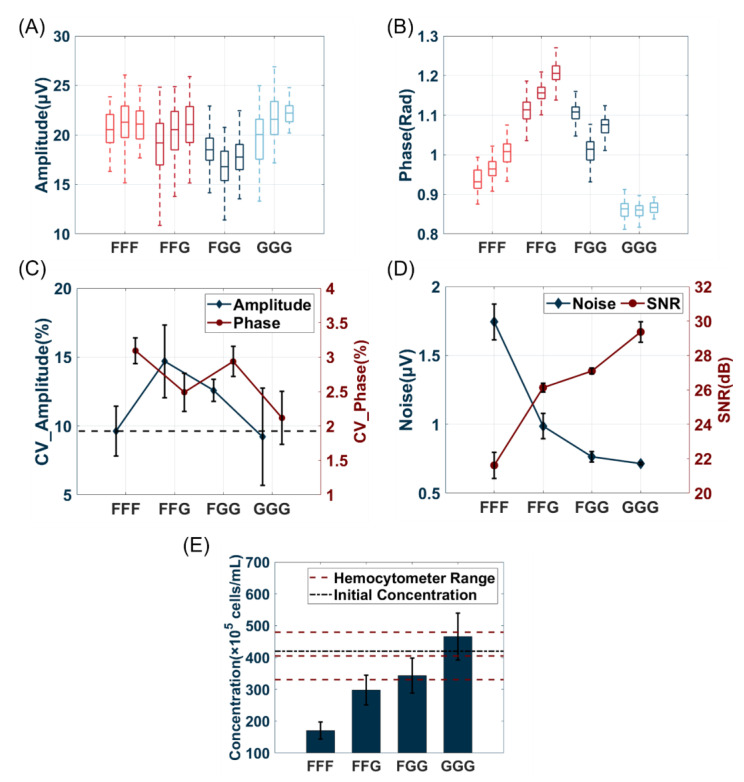
Comparison of diverse area of grounding electrode. Box plot of three repeated experiments about amplitude (**A**) and phase (**B**). (**C**) The CV of bead events of amplitude and phase. (**D**) The average SNR of all events and Noise values. (**E**) The bead concentration is measured by MIC.

The channel size of this experiment was set the same as in Section 3.1. The grounding electrode layout was the most optimal among the three bypass states. Subsequently, the effect of the grounding electrode area was investigated. The grounding electrode area was changed by altering the number of electrodes (0, 1, 2, and 3 pairs, represented as the FFF, FFG, FGG, and GGG electrode layouts, respectively) connected to the grounding beside the detection electrodes. As shown in Figure 5A–C, no direct correlation was observed between the area of the grounding electrode and the CV for both amplitude and phase. However, the GGG electrode layout exhibited the most favorable results owing to its lowest CV of amplitude and phase, highest SNR, and lowest noise. As shown in Figure 5D, increasing the area of the grounding electrode improved the SNR and decreased the noise, thereby promoting a higher sensing sensitivity and enabling the detection of smaller particle signals. These experimental results were consistent with the simulation results, and the larger bypass grounding electrode had a better detection performance. Nevertheless, there are limitations in increasing the grounding area owing to fabrication difficulties and liquid leakage probability. The processed data are presented in Table S3.

The MIC system, which was developed based on the Coulter counter [36], requires high bio-particle detection sensitivity and precision. Owing to the non-uniform distribution of the electric field in the coplanar electrode layout within the channel, bio-particles passing through different height positions generate pulses with diverse amplitudes. When a bio-particle passes through a higher position in the channel, sparse electric field lines may cause small bio-particle events to be obscured by noise [15]. As shown in Figure 5E, an increase in the grounding electrode area led to an increase in both the number of detected beads and the estimated concentration. The concentration estimation results of FGG and GGG were in the range of the hemocytometer counting results, and the average value was nearly equal to the concentration calculated using the parameters provided by the manufacturer, indicating a higher detectable sensitivity. These results indicate that using a bypass grounding electrode can enhance the detected capability, and a larger area results in higher rates. The coefficients of channel loss k with the four electrode layouts were 0.579, 0.264, 0.152, and −0.151, respectively. A negative value of k can represent all particles that can be detected using the current channel and GGG electrode layout. Concentration errors may be caused by residual debris and beads during channel cleaning as well as differences in the liquid height at the inlet. Bead concentrations measured in the MIC system with different grounding electrode areas and hemocytometer detection results are provided in Section 3 of the Supplementary Materials.

### 3.3. The Sensitivity of Particle Volume Detection Using GGG Layout Electrode

**Figure 6:**
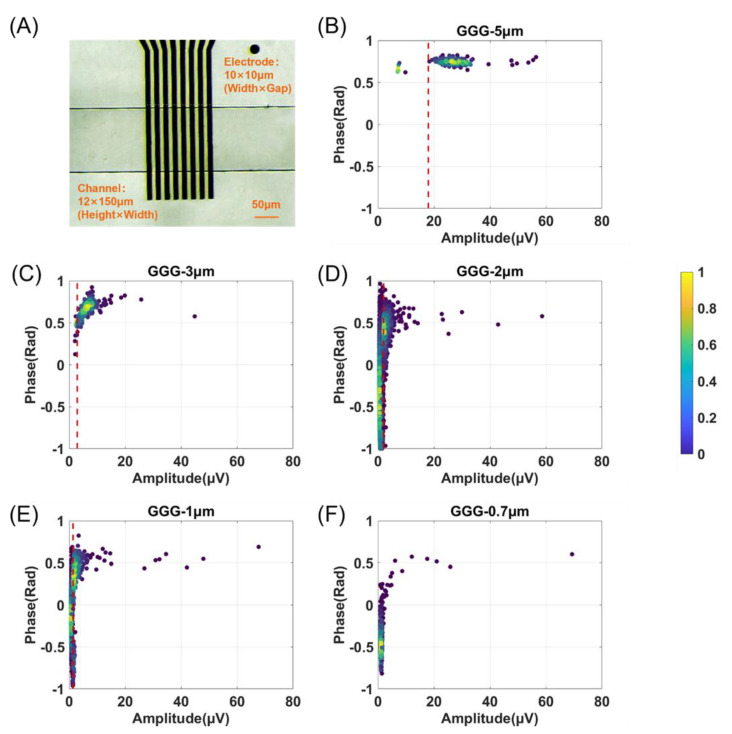
The sensitivity of particle detection volume. (**A**) Optical microscopy of the detection region captured using a CCD camera. Scatter plots of the amplitude and phase of different sizes of beads (**B**–**F**): Φ 5, 3, 2, 1, and 0.7 μm, respectively) detection using a GGG electrode layout and a wide channel of 12 × 150 μm (red dash lines represent the thresholds of each group; the color bar indicates the degree of clustering of scatter points).

It has been reported that better particle events can be measured in the shrinking detection region [31]. However, the liquid throughput is limited. By increasing the liquid throughput of the MIC chip, samples with lower particle concentrations can be analyzed in time without enrichment processes, such as the online detection of bacteria in tap water. Based on the information in Section 3.1 and Section 3.2, we fabricated a MIC device utilizing the GGG electrode layout with a wide channel (150 μm) for small particle detection (seen as Figure 6A). The detection sensitivity of the MIC system was estimated by using a bypass grounding electrode adjacent to the sensing electrode. The scatter plots of beads (Φ 5, 3, 2, 1, and 0.7 μm) detected were depicted in Figure 6B–F. This device can successfully detect Φ 3 and 5 μm beads, achieving independent and compact clusters. The particles of Φ 2 and 1 μm also can be detected, but their clusters are too close to the noise to be differentiated clearly. The sensitivity of particle volume (Φ 1 μm) detection reaches an impressive value of 0.00097% (compared to nanopore technology < 0.01% [35]). The protuberant amplitude of Φ 5, 3, 2, and 1 μm (the clusters exceed the threshold) was about 18, 2.9, 2.0, and 1.7 μV, respectively. Below the threshold was considered a noise event. On one hand, there is a clear amplitude-based dividing line; furthermore, if it is unclearly demarcated from noise due to its small size, a bumpy peak should appear in its phase profile. Since particles of similar material exhibit small variations in phase distribution, they are not widely dispersed across phases. The background noise randomly generated by electrical signals or fluid fluctuations will therefore be distributed throughout all phases. The result of Φ 0.7 μm beads demonstrates only a few events of ~0.5 rad; thus, the channel loss k is ~1, and Φ 0.7 μm cannot be recognized as a detectable size. The real-time videos of detecting Φ 2 and 1 μm beads can be seen in Videos S1 and S2.

## 4. Conclusions and Outlook

In this study, we designed an 8-electrode MIC device and investigated the impact of different bypass electrode configurations on the MIC system by electric field and experimental verification. The electric field simulation results were consistent with the experimental results. Our findings demonstrate that the bypass grounding electrode exhibits optimal performance among the bypass electrode states. Consequently, we also compared the influence of varying the area of the grounding electrode on system detection and ultimately determined that a larger grounding electrode yields improved sensing capabilities under this specific configuration. A full grounded electrode (GGG) achieved optimal detection effectiveness and sensitivity. Comparing the no bypass with the GGG configuration, an increase in the average SNR of all bead events (>10 dB), a reduction in baseline noise (>5.9 times), a decrease in the CV of bead events in both amplitude and phase (approximately two and three times), and a higher detection sensitivity were achieved.

Then, a MIC device using the GGG layout was fabricated to detect small particles (Φ 0.7, 1, 2, 3, and 5 μm) in a wide channel (150 μm) and shown to be capable of detecting Φ 1 μm beads. The sensitivity of particle detection was 0.00097%. This implies that the throughput of the liquid can be increased, and the detection time of the MIC system can be reduced. This electrode optimization technique does not change the spatial volume of the detection region or require dilution of the particle concentration to prevent coincident particle events in the detection region. Moreover, it can be applied as a general technique that can be adapted to various electrode and channel designs to optimize the detection of particles of any size. This enables a wide range of applications for the MIC system in single-cell analysis, such as monitoring bacterial levels in circulating water at space stations and analyzing apoptotic bodies, exosomes, extracellular vesicles, viruses, and macromolecular proteins.

## Data Availability

Data are contained within the article and Supplementary Materials.

## Supplementary Materials

The following supporting information can be downloaded at https://www.mdpi.com/article/10.3390/bios14040204/s1. Figure S1: Conventional detection electrode layout (no bypass) was captured by a CCD camera. Figure S2: The electric field simulation of the three bypass states with an 8-electrode MIC chip: (A) no bypass, (B) FFF, (C) GGG. Figure S3: Simulation of equipotential planes. (A) No bypass electrode layout. (B) Floating electrode layout. When 50 equipotential planes with the same drop were set up in COMSOL, it can be observed that the electric potential of the bypass floating electrode was more concentrated than that of the no bypass electrode. Figure S4: The electric field simulation of the different bypass floating electrode areas (the electrode length is the same, but the width is different): (A) 10 μm, (B) 20 μm, (C) 30 μm. Table S1: Impedance data for no bypass and FFF electrode layout (electrode: 10 × 10 μm (width × gap), channel: 10 × 10 × 12 μm (length × width × height)). Table S2: Impedance data for FFF and GGG electrode layout (electrode: 10 × 10 μm (width × gap), channel: 10 × 10 × 12 μm (length × width × height)). Table S3: Impedance data for diverse width of grounding electrode (electrode: 10 × 10 μm (width × gap), channel: 10 × 10 × 12 μm (length × width × height)). Vedio 1: 2 um-GGG. Vedio 2: 1 um-GGG.
