# Supplementary material for: Optimizing Microfluidic Impedance Cytometry by Bypass Electrode Layout Design
Source: Biosensors (Basel). 2024 Apr 19;14(4):204. doi: 10.3390/bios14040204 (PMC11048680; doi:10.3390/bios14040204)
Supplement: Supplementary file 1 [file biosensors-14-00204-s001.zip › biosensors-2912586-supplementary.pdf]

Supplementary Materials

Optimizing Microfluidic Impedance Cytometry by Bypass Electrode Layout Design

Guangzu Wu [1,2], Zhiwei Zhang [1,2], Manman Du [3], Dan Wu [4], Junting Zhou [4], Tianteng Hao [4] and Xinwu Xie [1,2,*]

[1] Systems Engineering Institute, Academy of Military Sciences, People's Liberation Army, Tianjin 300161, China
[2] National Bio-Protection Engineering Center, Tianjin 300161, China
[3] School of Environmental Science and Engineering, Tianjin University, Tianjin 300072, China
[4] School of Electronic Information and Automation, Tianjin University of Science and Technology, Tianjin 300222, China

* Correspondence: xinwuxie@163.com

1 Bypass electrode layout

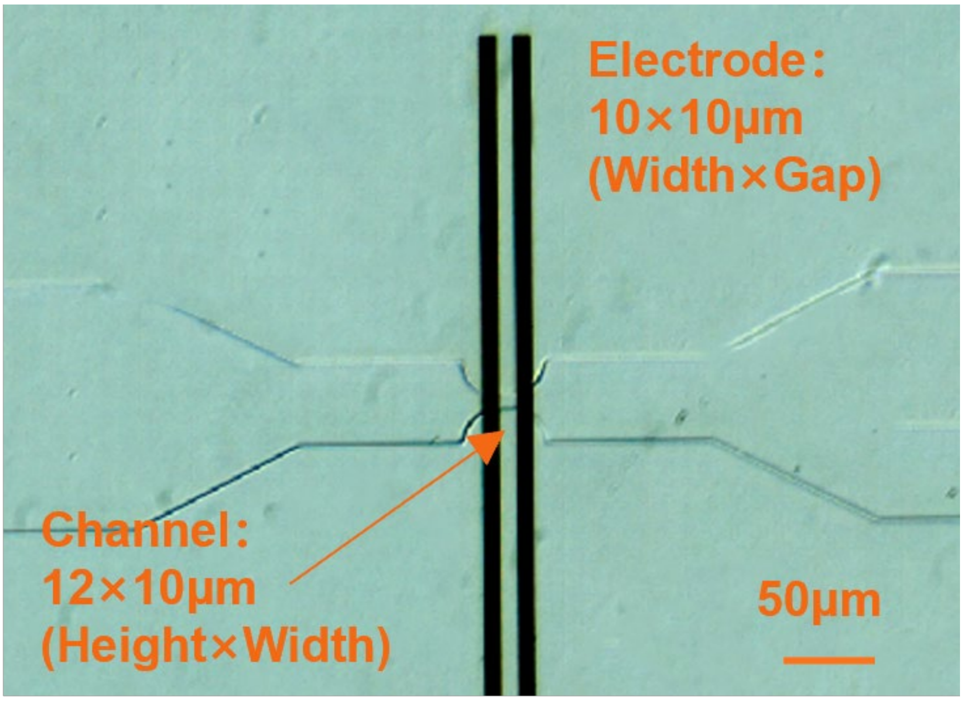

Figure S1. Conventional detection electrode layout (no bypass) was captured by a CCD camera.

2 Electric field simulation

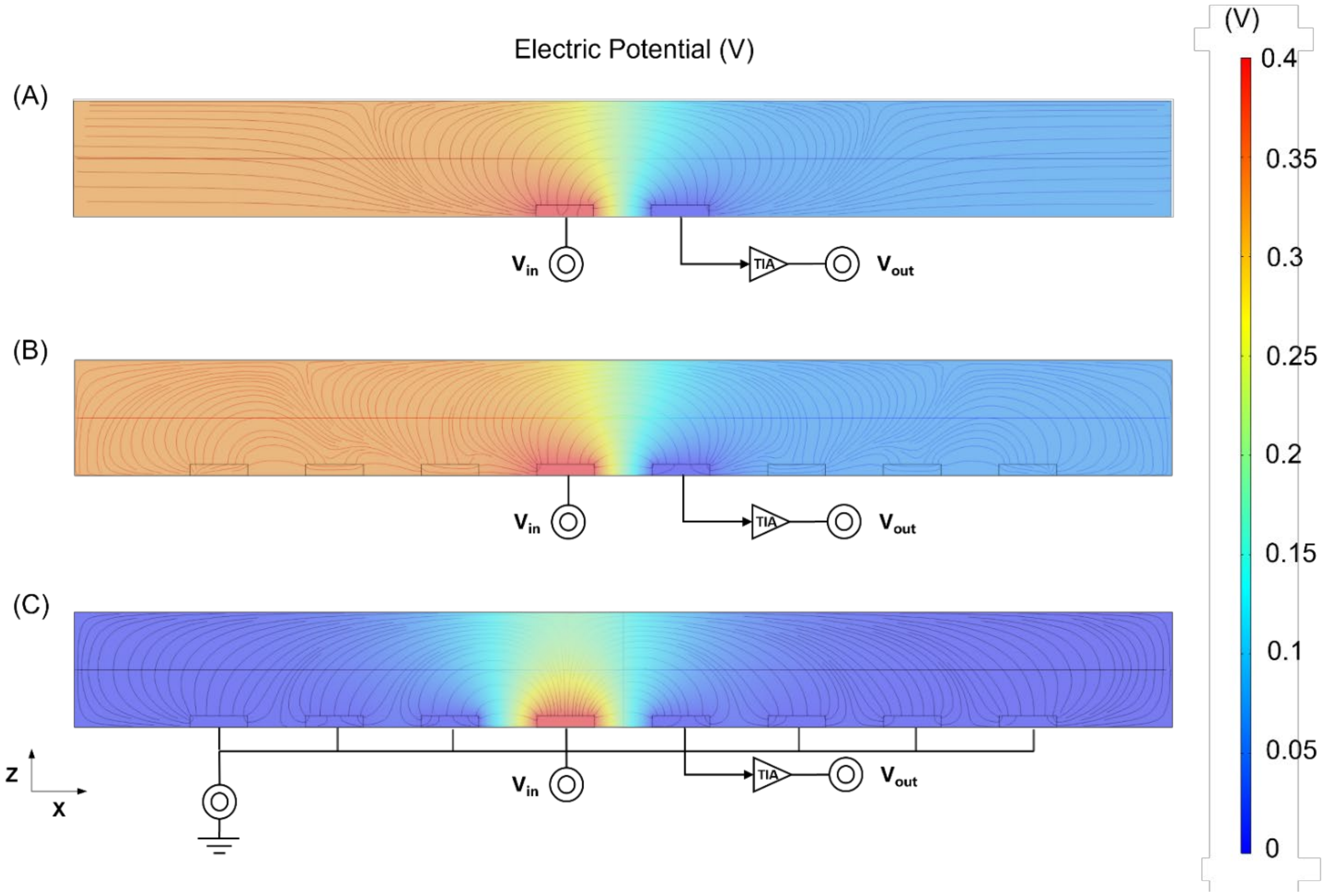

Figure S2. The electric field simulation of the three bypass states with an 8-electrode MIC chip: (A) no bypass, (B) FFF, (C) GGG.

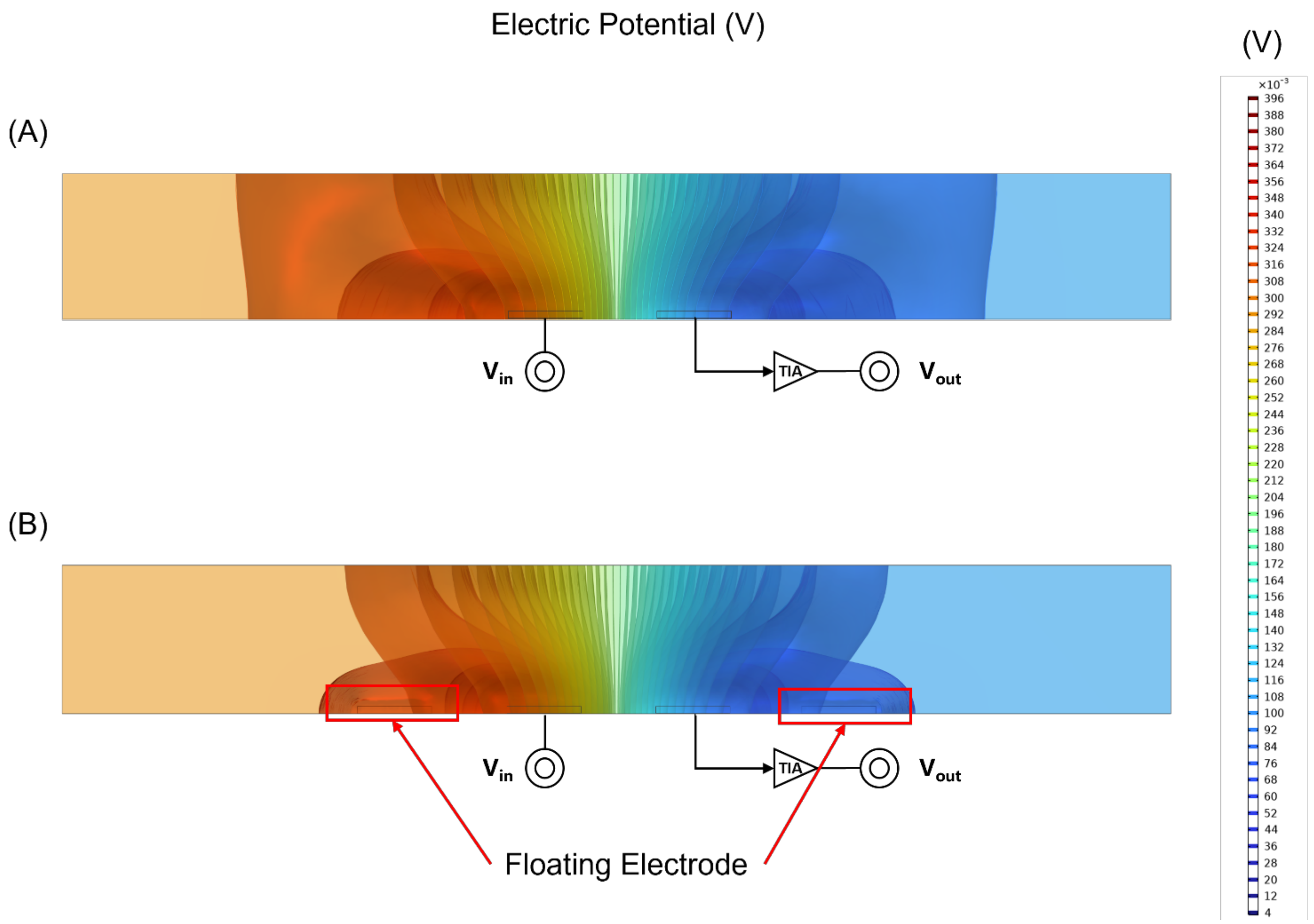

Figure S3. Simulation of equipotential planes. (A) No bypass electrode layout. (B) Floating electrode layout.

When 50 equipotential planes with the same drop were set up in COMSOL, it can be observed that the electric potential of the bypass floating electrode was more concentrated than that of the no bypass electrode.

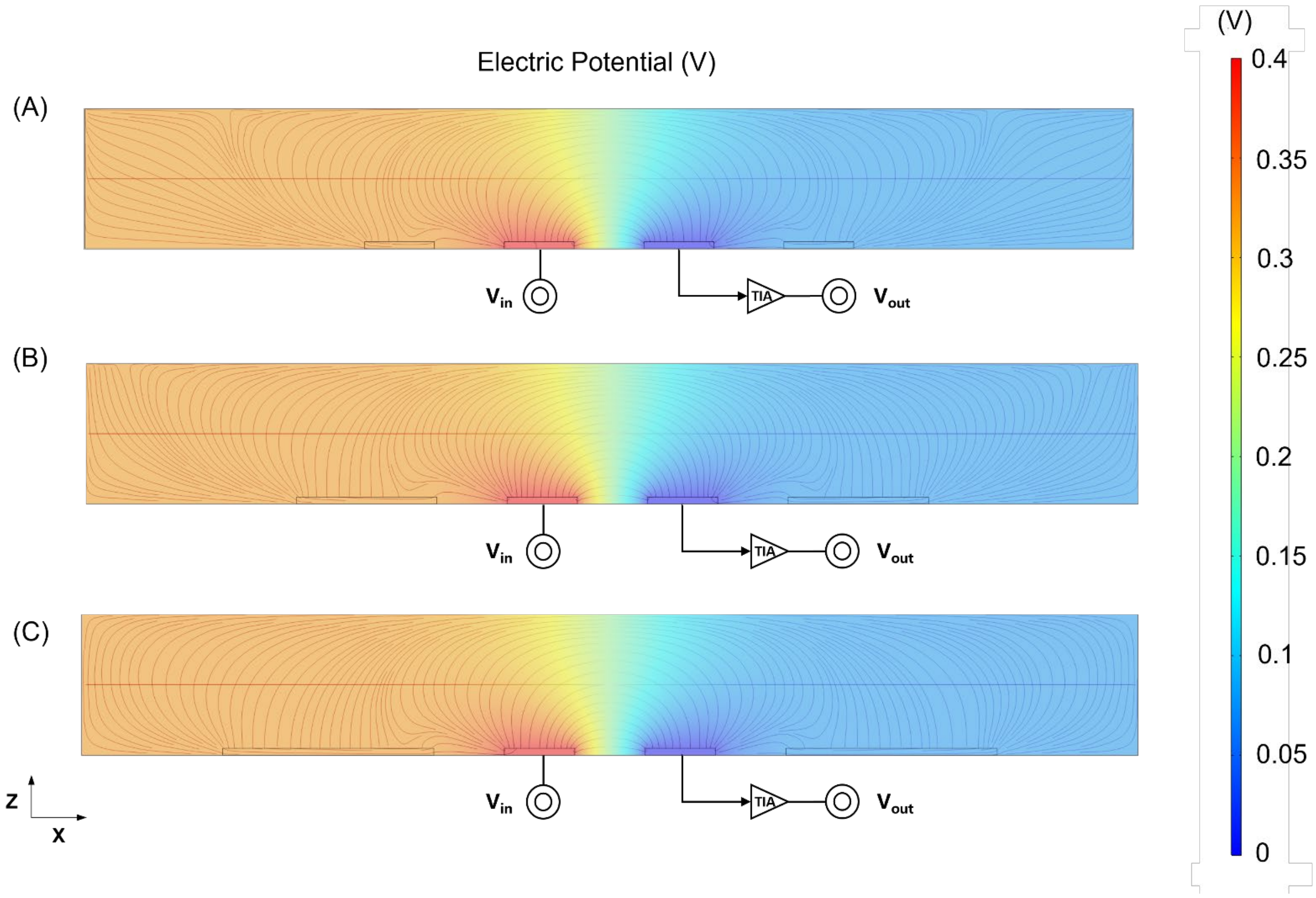

Figure S4. The electric field simulation of the different bypass floating electrode areas (the electrode length is the same, but the width is different): (A) 10 μm, (B) 20 μm, (C) 30 μm.

3 Results were calculated from raw data

Table S1. Impedance data for **no bypass and FFF electrode layout** (electrode: 10 × 10 μm (width × gap), channel: 10 × 10 × 12 μm (length × width × height))

Electrode Layout	Amplitude (μV)	CV of Amplitude (%)	Detection Throughput (cells/mins)	SNB (dB)	Phase (Rad)	CV of Phase (%)	Noise (μV)
No Bypass1	32.59 ± 6.35	19.48	13.48	17.81	0.88 ± 0.075	8.51	4.11
No Bypass2	32.26 ± 6.03	18.69	11.43	17.66	0.82 ± 0.051	6.22	4.14
No Bypass3	32.56 ± 6.28	19.03	15.57	17.44	0.92 ± 0.044	4.73	4.34
FFF1	20.63 ± 1.79	8.67	14.60	20.77	0.94 ± 0.030	3.17	1.88
FFF2	21.15 ± 2.48	11.72	11.89	22.23	0.96 ± 0.028	2.88	1.62
FFF3	21.12 ± 1.79	8.49	16.18	21.85	1.01 ± 0.033	3.23	1.73

[1]**Notes:** The CVs are obtained by processing the original data; No Bypass: there is no bypass electrode except input and output working electrode pair; F: the bypass electrode is floating; G: the bypass electrode is connected to the ground. **(Same as in the remaining tables.)**

CV(%)=Standard deviation/Mean

Detection Throughput (cells/mins)=Particle_number (cells)/Times (mins)

$SNB(dB)=20*\log_{10}(Mean_{Amplitude}(V))/Noise(V)$

$Noise=\sqrt{(3*\sigma_{Z_{RE}})^2+(3*\sigma_{Z_{IM}})^2}$

Table S2. Impedance data for **FFF and GGG electrode layout** (electrode: 10 × 10 μm (width × gap), channel: 10 × 10 × 12 μm (length × width × height))

Electrode Layout	Amplitude (μV)	CV of Amplitude (%)	Detection Throughput (cells/mins)	SNB (dB)	Phase (Rad)	CV of Phase (%)	Noise (μV)
FFF1	20.63 ± 1.79	8.67	14.60	20.77	0.94 ± 0.030	3.17	1.88
FFF2	21.15 ± 2.48	11.72	11.89	22.23	0.96 ± 0.028	2.88	1.62
FFF3	21.12 ± 1.79	8.49	16.18	21.85	1.01 ± 0.033	3.23	1.73
GGG1	19.53 ± 2.18	11.17	25.50	28.72	0.86 ± 0.021	2.46	0.707

GGG2	21.83 ± 2.47	11.33	29.00	29.50	0.86 ± 0.019	2.19	0.726
GGG3	22.20 ± 1.14	5.13	29.50	29.88	0.87 ± 0.015	1.70	0.711

Table S3. Impedance data for **diverse width of grounding electrode** (electrode: 10 × 10 μm (width × gap), channel: 10 × 10 × 12 μm (length × width × height))

Electrode Layout	Amplitude (μV)	CV of Amplitude (%)	Detection Throughput (cells/mins)	SNB (dB)	Phase (Rad)	CV of Phase (%)	Noise (μV)
FFF1	20.63 ± 1.79	8.67	14.60	20.77	0.94 ± 0.030	3.17	1.88
FFF2	21.15 ± 2.48	11.72	11.89	22.23	0.96 ± 0.028	2.88	1.62
FFF3	21.12 ± 1.79	8.49	16.18	21.85	1.01 ± 0.033	3.23	1.73
FFG1	18.78 ± 3.33	17.73	26.55	26.42	1.11 ± 0.030	2.71	0.881
FFG2	20.58 ± 2.76	13.40	25.00	25.93	1.16 ± 0.025	2.18	1.03
FFG3	21.16 ± 2.74	12.94	26.54	26.04	1.21 ± 0.031	2.58	1.05
FGG1	18.60 ± 2.36	12.70	27.00	27.19	1.11±0.035	3.19	0.807
FGG2	16.67 ± 2.22	13.33	31.75	26.90	1.01±0.029	2.82	0.746
FGG3	17.72 ± 2.08	11.73	33.50	27.19	1.07±0.030	2.80	0.739
GGG1	19.53 ± 2.18	11.17	25.50	28.72	0.86 ± 0.021	2.46	0.707
GGG2	21.83 ± 2.47	11.33	29.00	29.50	0.86 ± 0.019	2.19	0.726
GGG3	22.20 ± 1.14	5.13	29.50	29.88	0.87 ± 0.015	1.70	0.711

4 Calculation of concentration

The **actual detection beads concentration** of the FFF layout is (0.0152~0.0190) × 10^9 cells/mL.

The **actual detection beads concentration** of the FFG layout is (0.0265~0.0331) × 10^9 cells/mL.

The **actual detection beads concentration** of the FGG layout is (0.0305~0.0382) × 10^9 cells/mL.

The **actual detection beads concentration** of the GGG layout is (0.0414~0.0518) × 10^9 cells/mL.

According to the results of the **hemocytometer calculation**, the concentration of the bead suspension medium (Φ 5 μm) to be measured was calculated three times, and the results were 0.0375 × 10^9 cells/mL, 0.035 × 10^9 cells/mL, and 0.049 × 10^9 cells/mL, respectively. And the average concentration of the hemocytometer is 0.0405 × 10^9 cells/mL.

The average concentration is 0.0177 × 10^9, 0.0298 × 10^9, 0.03435 × 10^9, and 0.0466 × 10^9 cells/mL for FFF, FFG, FGG, and GGG, respectively.
